# Pre-sleep arousal as a possible mechanism driving sleep problems in relation to ADHD traits

**DOI:** 10.1038/s41598-025-09866-3

**Published:** 2025-07-08

**Authors:** Daniel Smullen, Tamar Kolodny, Andrew P. Bagshaw, Carmel Mevorach

**Affiliations:** 1 Centre for Human Brain Health and School of Psychology, University of Birmingham, Birmingham, UK; 2 Department of Psychology, School of Brain Sciences and Cognition, Ben-Gurion University of the Negev, Beer Sheva, Israel

**Keywords:** Psychology, Signs and symptoms

## Abstract

Attention Deficit Hyperactivity Disorder (ADHD) is inextricably linked with sleep problems. Clinically diagnosed poor sleep is common in ADHD, and subclinical sleep difficulties are frequently reported. However, the mechanisms which underly poor sleep in ADHD are poorly understood. Since one of the most consistently reported sleep difficulties is sleep initiation, it is plausible that pre-sleep arousal plays an important role, but this has not been confirmed. We investigated this by assessing neurotypical adult participants’ self-reported perceptions of their sleep difficulties and pre-sleep arousal in relation to their ADHD-like traits. We acquired two independent datasets: an online dataset (*n* = 104) and an in-person replication dataset (*n* = 96). We first assessed the association between ADHD-like traits and sleep quality generally, as well as sleep-onset problems specifically. We then tested the extent to which pre-sleep arousal mediated the association between ADHD traits and sleep initiation problems. There was a clear association in both datasets between ADHD-like traits and their sleep quality – high expression of ADHD-like behaviours coincided with reduced sleep quality. This relationship was largely mediated by the levels of pre-sleep arousal: the link between ADHD-like traits and sleep quality was explained by the relationship between ADHD and (primarily cognitive) pre-sleep arousal. Our findings highlight the role of pre-sleep arousal in the relationship between ADHD-like traits and sleep quality, which could direct future interventions.

**Supplementary Information:**

The online version contains supplementary material available at 10.1038/s41598-025-09866-3.

## Introduction

Attention deficit hyperactivity disorder (ADHD) is a neurodevelopmental condition which affects roughly 2.5% of adults^1,2^. ADHD often co-occurs with other psychiatric and medical conditions, with one of the most prevalent co-occurring issues being sleep problems. Although these are not typically screened for in a diagnostic setting, studies of sleep in ADHD populations reveal that sleep problems are reported by 70–90% of individuals^3,4^. These sleep problems manifest in increased prevalence of several clinically diagnosed sleep disorders in ADHD, including insomnia disorders associated with initiating and maintaining sleep, sleep-wake transition disorders, disorders of excessive somnolence and sleep hyperhidrosis^5^. Similar sleep problems in ADHD are self-reported even without a clinical diagnosis of a sleep disorder^6–10^. Those with ADHD are also more likely to have an evening chronotype than their neurotypical peers^11–15^. Evening chronotypes (also known as ‘night owls’) are more likely to suffer from disrupted sleep and are more likely to be diagnosed with a sleep disorder^16,17^.

One of the most frequently reported sleep difficulties in people diagnosed with ADHD is in falling asleep, or sleep initiation^18^. In people with insomnia, sleep initiation problems have been attributed to both cognitive (related to mental activity) and somatic (related to physiological activity) pre-sleep arousal, although cognitive arousal has been suggested to be up to ten times more frequently experienced than somatic arousal^19^. For example, measures of sleep onset latency from actigraphy correlate with self-reported pre-sleep cognitive activity in people with insomnia^20^. The relevance of pre-sleep arousal to sleep-onset latency has also been demonstrated in good sleepers, where increasing pre-sleep cognitive arousal is associated with delayed sleep latency^21,22^. It is therefore plausible that if pre-sleep arousal drives sleep-onset issues in people with insomnia and typical sleepers, it may also drive sleep-onset issues in ADHD. Determining what role pre-sleep arousal plays in ADHD-related sleep issues may guide interventions aimed to improve these sleep problems.

Currently, the underlying mechanisms for impaired sleep in ADHD remain unclear. Understanding these mechanisms, what those with impaired sleep experience and what they attribute their sleep impairment to, could provide new directions for future research into the neurological underpinnings of ADHD-impaired sleep and, importantly, for its treatment. Treating impaired sleep in ADHD may also lead to benefits across other facets of the condition. This is because, irrespective of ADHD, poor sleep is associated with cognitive impairments often seen in ADHD. For instance, performance in sustained attention and response inhibition are both impaired by poor sleep^23–27^. As deficits in these cognitive functions are core characteristics of ADHD^28–30^ they might be exacerbated by co-occurring sleep problems, and hence better understanding of sleep issues in ADHD may provide a potential pathway to improve cognitive function in the disorder.

The present study investigated ADHD-traits, pre-sleep arousal, and sleep in a general population of university students, as opposed to a clinically diagnosed ADHD cohort. An advantage of studying ADHD-traits in the general population is that they still exist in varying degrees whilst recruitment difficulty is reduced, allowing for recruitment of a larger sample, and at the same time reducing the impact of ADHD medications and sleep medications as confounding variables. Investigating ADHD-traits in the general population also addresses the issue that psychopathological symptoms are continuous rather than categorical. This mitigates the limitations of traditional taxonomies of neurodevelopmental disorders surrounding the somewhat arbitrary boundaries between what is classed as disorder and normality when the disorder is itself referred to as a spectrum^31,32^. ADHD symptoms are reported at greater than average rates in university students, with as many as 8% of students reporting clinically significant levels of symptoms^33^. There are a range of possible explanations for these heightened levels, including the effect of academic pressure and lifestyle factors such as alcohol usage^34,35^. Whatever the reasons for their over-representation in university student samples, this provides a way of studying the relationships between ADHD traits and sleep without some of the complications that arise from clinical samples.

In the present study we investigated the role of pre-sleep arousal in sleep problems in ADHD-traits by assessing participants’ perceptions of their sleep difficulties and pre-sleep arousal at the same time as their ADHD-like symptomatology. We conducted our analysis on two independent datasets, the first being a dataset collected online during the UK lockdown, and the second – a replication dataset - collected in-person a year later after lockdown restrictions were lifted to ensure our findings were not confounded by online data collection, and to enhance the reliability of our results. In both cohorts we first assessed the association between ADHD-like traits and sleep quality generally. We then assessed the association between ADHD symptoms, as well as sleep problems, with cognitive and somatic pre-sleep arousal. Finally, we tested the extent to which pre-sleep arousal mediated the association between ADHD and sleep problems. In a further exploratory analysis, we tested which specific elements of pre-sleep arousal best predicted difficulties in falling asleep.

## Methods

### Participants

Participants in both the online dataset (collected during the 2021/2022 academic session, during the COVID-19 pandemic) and the replication dataset (collected in-person during the 2022/2023 academic session) were Psychology students at the University of Birmingham, with no overlap between the two samples. The replication dataset was collected for pure replication purposes, as well as to ensure the findings were not driven by the pandemic and its possible effects on mental health, attention and sleep. Participants who reported a previous clinical ADHD or sleep disorder diagnosis were excluded in order to ensure that the effects of ADHD medications and sleep medications were limited in our datasets.

The first dataset was collected online and included 110 adults (24 males and 86 females, mean age(SD) = 19.41(1.30)y). Following outlier removal (as described below) there remained 104 participants (18 males and 86 females, mean age(SD) = 19.36(1.29)y). The second replication dataset was collected in-person in the lab on year later, and included 98 adults (19 males and 79 females, mean age(SD) = 19.34(1.05)y). Following outlier removal there remained 96 participants in the replication study (19 males and 77 females, mean age(SD) = 19.35(1.05)y).

All methods used in the study conformed to the Declaration of Helsinki and was approved by the STEM ethics committee at the University of Birmingham. All participants provided informed consent at the beginning of the study and were informed that they could withdraw their participation and data at any point during or within 5 weeks of the experiment. Participants were compensated for their participation with course credits.

### Materials

The study utilised a series of self-report questionnaires to assess ADHD symptomatology on the one hand and sleep problems on the other. The final step of our analysis of the online dataset was a mediation analysis which investigated how pre-sleep arousal mediates the relationship between ADHD symptom severity and sleep quality. Only those measures implicated in the mediation analysis of the online dataset were then used in the replication dataset.

#### Sleep quality measures

Sleep quality has numerous facets which are typically assessed using different questionnaires to ensure they are measured comprehensively. As such, we deployed two commonly used questionnaires which assess various subjective elements of sleep quality and severity of insomnia-related sleep issues.

#### Pittsburgh sleep quality index (PSQI)

The PSQI assesses self-reported sleep quality^36^. It consists of 20 questions which feed into seven components of sleep quality: subjective sleep quality, sleep latency, sleep duration, sleep efficiency, sleep disturbance, use of sleep medication and daytime dysfunction. Sleep quality is scored across each component and in a combined overall score. Higher scores suggest reduced sleep quality: Internal consistency for the overall PSQI score was α = 0.608 in the exploratory dataset and α = 0.600 in the replication dataset, with previously reported values ranging from α = 0.70 to 0.83^37^.

#### Insomnia severity index (ISI)

The ISI assesses the severity of daytime and night time insomnia symptoms and is often used clinically during the diagnostic process for the disorder^38^. It uses seven questions responded to via Likert scales to produce an overall insomnia symptom severity. It also provides guidance on categorising scores: 0–7 no clinically significant insomnia, 8–14 subthreshold insomnia, 15–21 clinical insomnia (moderate severity), 22–28 clinical insomnia (severe). Internal consistency for the overall ISI score was α = 0.825 in the exploratory dataset and α = 0.631 in the replication dataset, with previously reported values ranging from α = 0.65 to 0.92^39^.

#### Pre-sleep arousal measures

Two questionnaires were used to assess pre-sleep arousal. First, the Pre-Sleep Arousal Scale was used, which covers different domains and thus is an effective tool to study overall pre-sleep arousal elements. In addition, the Glasgow Cognitive Intrusions Index was included, specifically for the purpose of assessing individual pre-sleep thought content in a stepwise regression analysis where pre-sleep arousal contents were potential predictors (see below).

#### Pre-Sleep arousal scale (PSAS)

The PSAS is a 16-item questionnaire assessing the severity of experience of cognitive and physiological arousal when attempting to sleep^40^. The PSAS can be split into two components: somatic and cognitive pre-sleep arousals. Participants respond using Likert scales to report how much they experience arousals such as “shortness of breath, laboured breathing” [somatic] and “worry about falling asleep” [cognitive]. Higher scores reflect more severe experience. Subtypes of the PSAS reflect issues common in ADHD. Internal consistency for the overall PSAS score was α = 0.905 in the exploratory dataset and α = 0.862 in the replication dataset with previously reported values ranging from α = 0.82 to 0.91^41^. Internal consistency for the cognitive component of the PSAS were α = 0.899 in the exploratory datasets and α = 0.881 in the replication dataset, with previously reported values ranging from α = 0.67 to 0.94^41^. Internal consistency for the somatic component of the PSAS were α = 0.794 in the exploratory datasets and α = 0.651 in the replication dataset, with previously reported values ranging from α = 0.62 to 0.92^41^.

#### Glasgow cognitive intrusions index (GCTI)

The GCTI was designed to assesses the content, character and intrusiveness of cognitions during sleep onset in insomnia patients^42^. Likert scales are used to assess the frequency with which 25 thoughts, such as “your health”, have kept the participant awake over the previous week. Higher scores indicate higher frequency and hence more severe experience. Internal consistency for the overall GCTI score was α = 0.908 in the exploratory dataset, with the originally reported internal consistency score being α = 0.87^42^.

#### Adult ADHD Self-Report scale (ASRS)

The ASRS is a widely used questionnaire, which assess the severity with which an adult experiences ADHD symptoms^43^. It consists of 18 questions covering two components equally: inattention symptoms and hyperactivity/impulsivity symptoms, each corresponding to one item in the symptoms checklist for ADHD in the DSM. Participants rate how frequently they experience symptoms using a Likert scale. This provides a severity score ranging from 9 to 45 for both components, and a combined severity score ranging from 18 to 90. Higher scores reflect higher symptom severity. The ASRS is used as a pre-screening tool and does not have an overall cutoff in the traditional sense, but rather suggests that a patient warrants further testing if they score above a certain threshold on a certain number and combination of individual items. Internal consistency for the overall ASRS score was α = 0.747 in the exploratory dataset and α = 0.381 in the replication dataset, with previously reported values ranging from α = 0.63 to 0.72^44^.

### Experimental procedure

For the online dataset, participants were recruited via the University of Birmingham’s participant pool recruitment website (Sona; https://birmingham.sona-systems.com) and completed the entire experiment using their own PC in their own home. Once recruited, participants were redirected to Qualtrics (https://www.qualtrics.com), where they provided informed consent before completing the questionnaires outlined above in the same order (with the exception of GCTI which was completed before the PSAS). Foil questions were also used throughout the survey to ensure proper engagement with the questions. Foil questions appeared at a glance to fit with neighbouring questions but instead asked questions with obvious and objective answers (e.g. “what number comes after 5?”) to ensure that participants were reading the questions properly before answering. Any participants who answered the foil questions incorrectly would have been removed, although in practice all were answered correctly. Once the questionnaires were completed, participants completed a Go/No-go task which is not reported in this paper due to poor task engagement. Finally, they were debriefed and then compensated with course credits. The experiment was conducted completely online and without an experimenter present.

For the replication dataset, participants were again recruited via the University of Birmingham’s participant pool recruitment website (Sona), but completed the entire experiment in the lab. Participants were first directed to Qualtrics, where they provided informed consent before completing the questionnaires, in the same order as above, with only a subset of the questionnaires which were implicated in our mediation analysis being included in the analysis of the replication dataset: ASRS, ISI and PSAS. Foil questions were also used throughout the survey to ensure proper engagement with the questions. No participants answered these questions incorrectly. Finally, participants were debriefed and compensated with course credits. The experimenter was present in the lab, but at a distance, throughout the session.

### Outlier detection and statistical analysis

Statistical analyses were carried out in SPSS 26 (IBM SPSS Statistics, Version 26.0, https://www.ibm.com/products/spss-statistics). Outliers were determined using z-scores relative to the current sample on each questionnaire separately, using a +/- 3 standard deviations threshold. Outliers on questionnaire scores resulted in only that questionnaire being removed from further analysis. From the online dataset four participants were removed due to not meeting the inclusion criteria, whilst two were removed from the replication dataset. Also, in the online dataset, due to a technical failure resulting in missing datafiles only 83 participants had full PSQI data.

We tested the correlations between sleep quality measures (PSQI and ISI) and ADHD symptom severity; between sleep quality measures (PSQI and ISI) and pre-sleep arousal (PSAS and GCTI); and between ADHD symptom severity and pre-sleep arousal (PSAS and GCTI). Correlations were tested using non-parametric Spearman’s rank correlational coefficients due to non-normal distribution of the data, as assessed using the Shapiro-Wilk test of normality^45^. The strength of difference between correlations was assessed using a Fisher’s r to z transformation.

We also used mediation analyses to test whether pre-sleep arousal mediated the relationship between ADHD symptom severity and sleep quality. After first testing the assumptions of mediation analyses, such as normality of residuals, Hayes’ PROCESS Macro within SPSS was used to examine the mediation effect of pre-sleep arousal on the relationship between ADHD symptom severity and insomnia symptom severity. The analysis included 5000 bootstrap samples to generate bias-corrected 95% confidence intervals for the indirect effect.

Finally, in order to determine which specific pre-sleep arousal content was most associated with problems in sleep initiation, we performed a stepwise regression analysis in the online dataset, to model which cognitive and somatic pre-sleep arousal components best predicted sleep onset-latency (PSQI component 2). We opted to use this sleep quality component as pre-sleep arousal’s impact is most likely to be on the time taken to fall asleep. Potential predictors included in the stepwise regressions were those components (single questions) of the two pre-sleep arousal questionnaires (PSAS & GCTI) whose correlations with PSQI component 2, reached 0.05 significance. We corrected for multiple comparisons using Benjamini and Hochberg’s Step-Up Procedure to control for False Discovery Rate (FDR)^46^. All significant relationships reported in our results survived multiple comparison correction.

## Results

### Comparability of the online and replication datasets

**Fig. 1:**
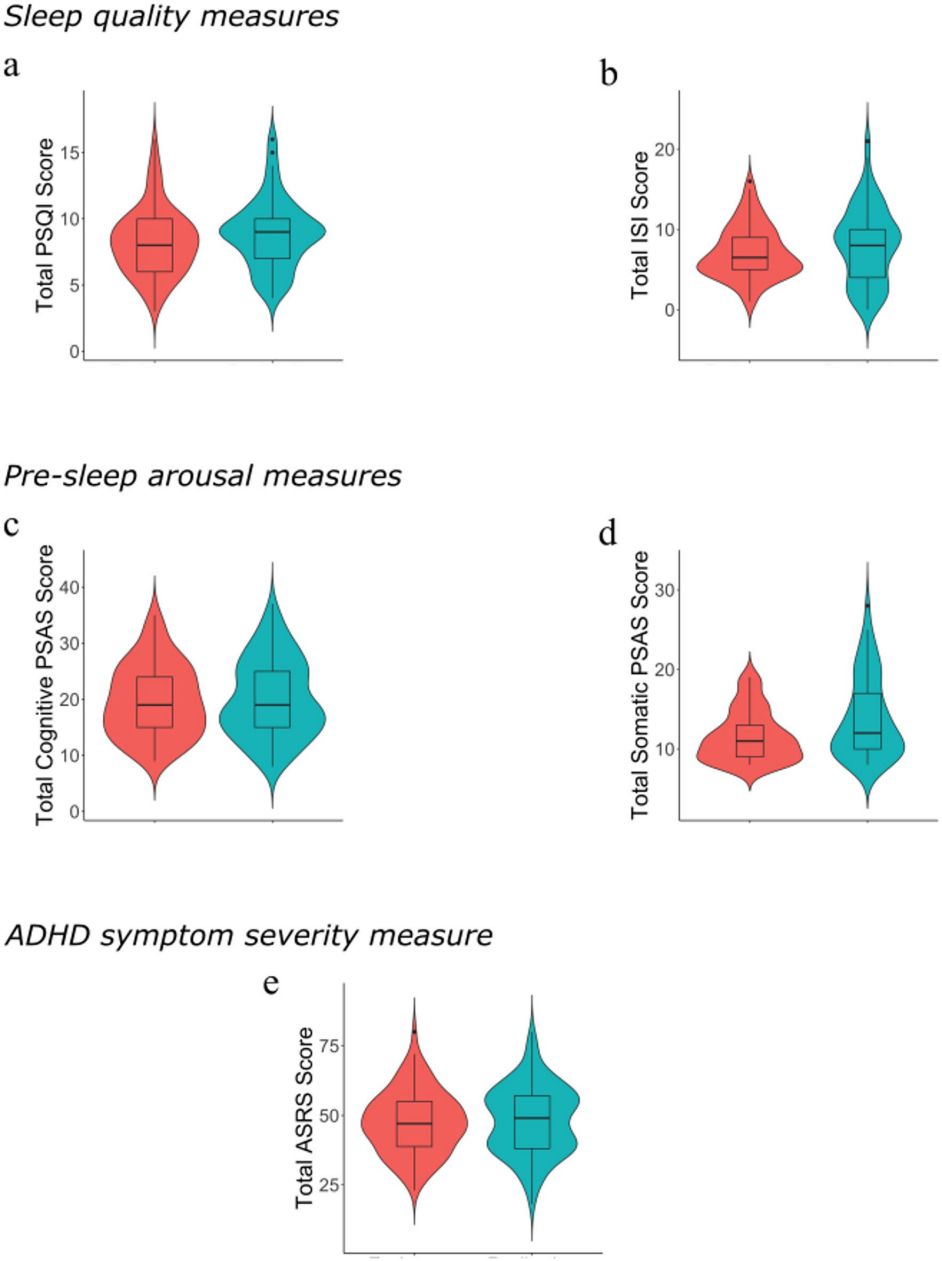
Violin plots depicting the differences between the online (red) and replication (blue) datasets for (**a**) total PSQI score, (**b**) total ISI score, (**c**) total cognitive pre-sleep arousal score, (**d**) total somatic pre-sleep arousal score and (e) total ASRS score.

Tables 1 and 2 provide an overview of the questionnaire data from the online and replication datasets, respectively. Figure 1 provides an overview of the distributions of total scores for the questionnaires. Figure 1 suggests that the two samples are generally comparable across all measures, as also indicated in Tables 1 and 2. Independent samples t-tests found that there was no significant difference between the online dataset’s ISI scores (mean = 8.12, sd = 4.75) and the replications dataset’s ISI scores (mean = 7.13, sd = 3.27): t(178) = 1.717, *p* =.088 (Fig. 1a). Likewise, there was no significant difference in total PSQI scores for the online (mean = 8.88, sd = 1.26) and replication (mean = 8.28, sd = 2.54) datasets: t(174) = 1.548, *p* =.123 (Fig. 1b), or for cognitive PSAS scores (online dataset (mean = 20.53, sd = 7.21), the replication dataset (mean = 19.77, sd = 6.44): t(196) = 0.365, *p* =.715 (Fig. 1c)). However, for somatic PSAS scores there was a significant difference between the online dataset (mean = 13.90, sd = 5.10) and the replication dataset (mean = 11.59, sd = 3.18): t(171) = 3.849, *p* <.001) (Fig. 1d). Lastly, there was no significant difference between the total ASRS scores for the online dataset (mean = 48.25, sd = 11.06) and the replication dataset (mean = 47.46, sd = 11.34): t(196) = 0.467, *p* =.641 (Fig. 1e).

**Table 1:** A summary of the questionnaire responses in the online dataset.

	n	Possible range	Obtained range	Mean	std	95% CI
PSQI	83	0–21	4–16	8.88	1.26	8.6–9.16
* M*	13		4–14	8.38	2.66	6.77–9.99
* F*	70		4–16	8.97	2.59	8.36–9.58
* Subjective sleep quality*	104	0–3	0–3	1.28	0.6	1.17–1.40
* M*	18		0–2	1.22	0.55	0.97–1.47
* F*	86		0–3	1.29	0.61	1.16–1.42
* Sleep latency*	104	0–3	0–3	2.78	0.42	2.70–2.86
* M*	18		0–3	2.72	0.46	2.51–2.93
* F*	86		2–3	2.79	0.41	2.70–2.88
* Sleep duration*	104	0–3	0–4	1.68	1.17	1.46–1.91
* M*	18		0–4	1.94	1.43	1.28–2.60
* F*	86		0–4	1.63	1.11	1.40–1.87
* Sleep efficiency*	84	0–3	0–3	0.87	0.94	0.67–1.07
* M*	13		0–1	0.62	0.51	0.34 − 0.90
* F*	71		0–3	0.92	0.10	0.90 − 0.94
* Sleep disturbance*	103	0–3	0–2	1.19	0.44	1.11–1.28
* M*	18		1–2	1.11	0.32	0.96–1.26
* F*	85		0–2	1.21	0.47	1.11–1.31
* Use of sleep medication*	104	0–3	0–3	0.08	0.39	0.01 − 0.16
* M*	18		0–0	0.00	0.00	0.00 − 0.00
* F*	86		0–3	0.09	0.42	0.00 − 0.18
* Daytime dysfunction*	104	0–3	0–3	1.45	0.81	1.29–1.61
* M*	18		0–3	1.44	0.92	1.02–1.87
* F*	86		0–3	1.45	0.79	1.28–1.62
ISI	101	0–28	0–21	8.12	4.75	7.19–9.05
* M*	17		0–16	6.53	4.58	4.35–8.71
* F*	84		0–21	8.44	4.75	7.42–9.46
GCTI	103	25–100	25–78	49.63	11.28	47.45–51.81
* M*	18		31–69	49.39	11.62	44.02–54.76
* F*	85		25–78	49.68	11.28	47.28–52.08
PSAS	102	16–80	16–65	34.32	11.01	32.18–36.46
* M*	17		18–56	36.18	12.73	30.13–42.23
* F*	85		16–65	33.95	10.76	31.66–36.24
* Cognitive*	103	8–40	8–37	20.53	7.21	19.14–21.92
* M*	18		10–35	20.61	7.68	17.06–24.16
* F*	85		8–37	20.01	6.82	18.56–21.46
* Somatic*	102	8–40	8–28	13.9	5.10	12.91–14.89
* M*	17		8–24	15.00	5.86	12.21–17.79
* F*	85		8–28	13.68	4.94	12.63–14.73
ASRS	102	18–90	18–80	48.25	11.06	46.10–50.40
* M*	18		30–64	47.72	10.12	43.05–52.40
* F*	84		18–80	48.36	12.77	45.63–51.09
* Inattention*	104	9–45	9–40	26.41	6.87	25.09–27.73
* M*	18		17–33	26.28	5.88	23.56–29.00
* F*	86		9–40	26.44	7.09	24.94–27.94
* Imp/Hyp*	102	9–45	9–40	21.76	6.6	20.48–23.04
* M*	18		13–32	21.44	5.07	19.10–23.72
* F*	84		9–40	21.83	6.90	20.35–23.31

Note: PSQI = Pittsburgh Sleep Quality Index; PSAS = Pre-sleep Arousal Scale; ISI = Insomnia Severity Index; GCTI = Glasgow Cognitive Thoughts Index; ASRS = Adult ADHD Self-Report Scale; Total PSQI data is reduced due to missing data for the sleep efficiency component; M = Male; F = Female.

**Table 2:** A summary of the questionnaire responses in the replication dataset.

	*n*	Possible range	Obtained range	Mean	std	95% CI
PSQI	93	0–21	3–16	8.28	2.54	7.76–8.80
* M*	17		4–15	9.06	3.13	7.57–10.55
* F*	76		3–16	8.11	2.38	7.56–8.65
* Subjective sleep quality*	94	0–3	0–3	1.16	0.61	1.04–1.28
* M*	18		1–3	1.44	0.62	1.15–1.73
* F*	76		0–3	1.09	0.59	0.96–1.22
* Sleep latency*	94	0–3	2–3	2.61	0.49	2.51–2.71
* M*	18		2–3	2.61	0.50	2.38–2.84
* F*	76		2–3	2.61	0.49	2.50–2.72
* Sleep duration*	94	0–3	0–3	1.36	0.93	1.17–1.55
* M*	18		0–3	1.61	1.09	1.11–2.11
* F*	76		0–3	1.30	0.88	1.10–1.50
* Sleep efficiency*	93	0–3	0–3	0.74	0.93	0.55 − 0.93
* M*	17		0–3	0.94	1.14	0.40–1.48
* F*	76		0–3	0.70	0.88	0.50 − 0.90
* Sleep disturbance*	94	0–3	0–2	1.14	0.48	1.04–1.24
* M*	18		1–2	1.33	0.49	1.10–1.56
* F*	76		0–2	1.09	0.47	0.98–1.20
* Use of sleep medication*	94	0–3	0–2	0.06	0.29	0.01 − 0.12
* M*	18		0–0	0	0	0–0
* F*	76		0–2	0.08	0.32	0.01 − 0.15
* Daytime * *dysfunction*	94	0–3	0–3	1.22	0.71	1.08–1.36
* M*	18		0–2	1.17	0.62	0.88–1.46
* F*	76		0–3	1.24	0.73	1.08–1.40
ISI	96	0–28	1–16	7.13	3.27	6.48–7.78
* M*	19		1–11	6.84	2.43	5.75–7.93
* F*	77		1–16	7.19	3.46	6.42–7.96
PSAS	95	16–80	17–53	31.42	8.48	29.72–33.13
* M*	19		19–44	31.32	7.44	27.98–34.67
* F*	77		17–53	31.44	8.76	29.48–33.40
* Cognitive*	95	8–40	9–35	19.77	6.44	18.48–21.07
* M*	19		10–31	19.95	5.70	17.39–22.51
* F*	76		9–35	19.72	6.64	18.23–21.21
* Somatic*	95	8–40	8–19	11.59	3.18	10.95–12.23
* M*	19		8–19	11.95	3.34	10.45–13.45
* F*	76		8–19	11.50	3.16	10.80–12.21
ASRS	96	18–90	23–80	47.46	11.34	45.19–49.73
* M*	19		31–80	45.53	11.28	40.46–50.60
* F*	77		23–72	47.94	11.38	45.40–50.48
* Inattention*	92	9–40	14–43	24.89	6.01	23.66–26.12
* M*	18		16–43	26.83	6.70	23.74–29.93
* F*	74		14–41	24.42	5.78	23.10–25.74
* Imp/Hyp*	94	9–40	9–39	22.30	6.56	20.97–23.63
* M*	19		10–34	21.61	7.46	18.26–24.96
* F*	76		9–39	22.46	6.37	21.03–23.89

Note: PSQI = Pittsburgh Sleep Quality Index; PSAS = Pre-sleep Arousal Scale; ISI = Insomnia Severity Index; ASRS = Adult ADHD Self-Report Scale; PSQI data is reduced due to missing data for the sleep efficiency component.

Overall, the data demonstrated considerable variability within datasets, consistent with the idea that studying traits as opposed to diagnoses is reasonable, but similar variability across datasets, suggesting it reasonable to employ the replication dataset in its intended function. We analysed the two datasets separately to preserve the integrity of each as independent samples and to assess the consistency of our findings across replication. While no significant differences were observed between the datasets (apart from somatic PSAS), they were collected independently with differences in recruitment methods and data collection conditions, making it important to evaluate them separately rather than merging them into a single analysis.

### The relationship between ADHD symptom severity and sleep measures

**Fig. 2:**
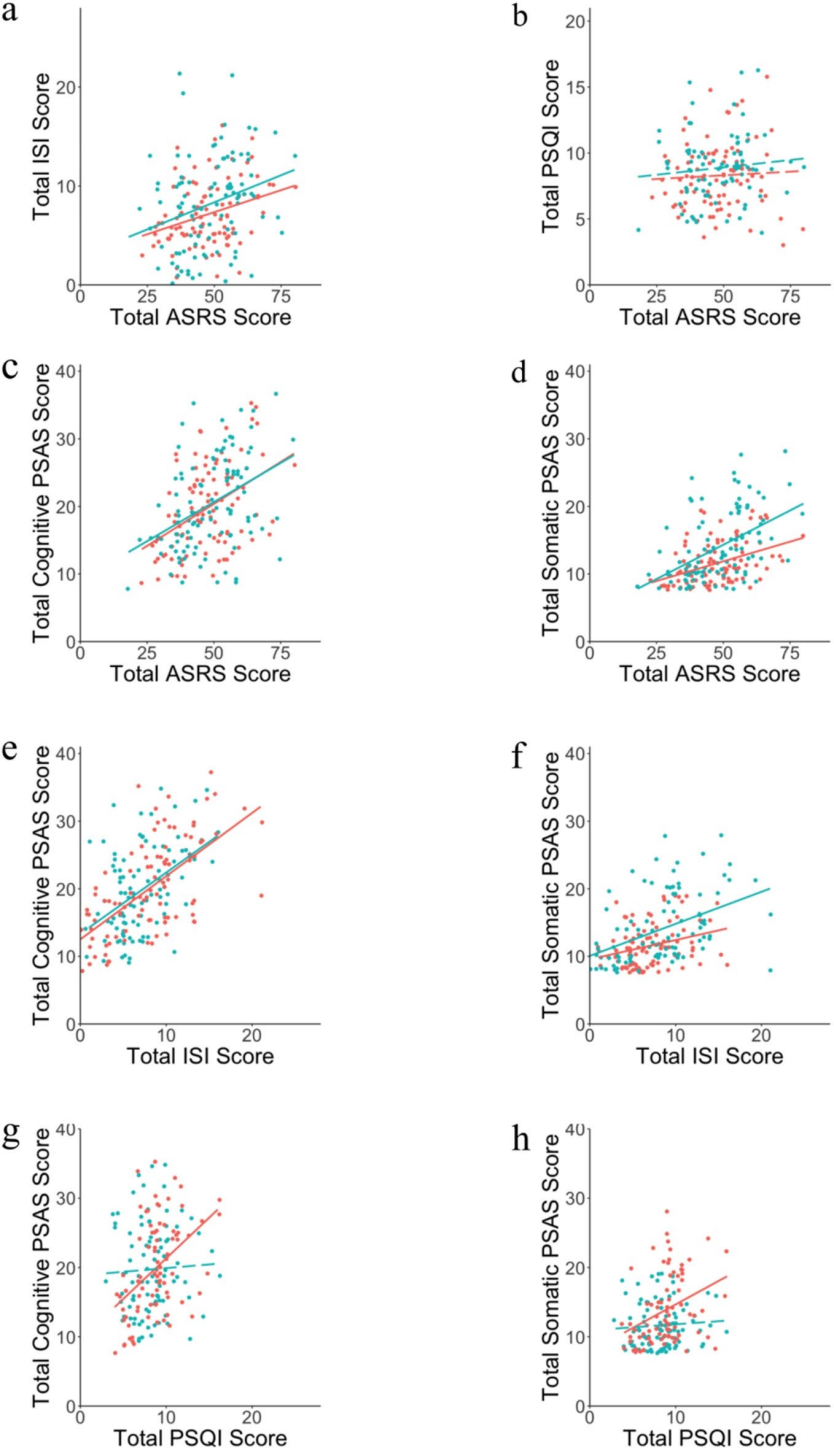
Jitterplots depicting the relationships for the online (red) and replication (blue) datasets between total ASRS score and (**a**) total ISI score, (**b**) total PSQI score, (**c**) total cognitive PSAS score, and (**d**) total somatic PSAS score, as well as between total ISI score and (**e**) total cognitive PSAS score, (**f**) total somatic PSAS score, and also between total PSQI score and (**g**) total cognitive PSAS score and (**h**) total somatic PSAS score. Significant relationships have a solid line of best fit.

We first assessed the relationship between the two sleep measures (overall sleep quality - PSQI scores, and Insomnia Severity - ISI scores) and ADHD symptom severity (ASRS scores). In the online dataset, we found that ADHD symptom severity (ASRS) significantly and positively correlated with insomnia symptom severity (ISI; r_s_(99) = 0.330, *p* =.003; Fig. 2a). However, we did not find a significant relationship between ADHD symptom severity and overall sleep quality as assessed by the PSQI (r_s_(81) = 0.077, *p* =.496; Fig. 2b). The same was found in the replication dataset: a significant association between ADHD symptom severity and insomnia symptom severity (r_s_(96) = 0.278, *p* =.006; Fig. 2a), but no significant relationship between ADHD symptom severity and overall sleep quality as assessed by the PSQI (r_s_(93) = − 0.102, *p* =.332; Fig. 2b). When controlling for the effect of sex, no difference was found in the outcomes of correlation tests (see supplementary Tables 1 and 2).

### The relationship between ADHD symptom severity and cognitive and somatic pre-sleep arousal

Next, we investigated the relationship between ADHD symptom severity (ASRS scores) and cognitive and somatic pre-sleep arousal (PSAS scores) using Spearman’s Rank Correlation Coefficients. For the online dataset, we found a statistically significant positive correlation between ADHD symptom severity and cognitive pre-sleep arousal (r_s_(100) = 0.405, *p* <.001; Fig. 2c) and ADHD symptom severity and somatic pre-sleep arousal (r_s_(101) = 0.529, *p* <.001; Fig. 2d). In the replication dataset, we also found a statistically significant positive correlation between ADHD symptom severity and cognitive pre-sleep arousal (r_s_(95) = 0.402, *p* <.001; Fig. 2c) and ADHD symptom severity and somatic pre-sleep arousal: r_s_(95) = 0.417, *p* <.001; Fig. 2d). As previous research in insomnia pointed to a greater prevalence of cognitive pre-sleep arousal relative to somatic pre-sleep arousal^19^ we tested for a difference in the strength of the association between ADHD symptoms and somatic vs. cognitive arousal using Fisher’s r to z transformation. However, there was no significant difference in the online dataset (*p* =.379) or the replication dataset (*p* =.452). When controlling for the effect of sex, no difference was found in the outcomes of correlation tests (see supplementary Tables 1 and 2).

### The relationship between pre-sleep arousal and sleep quality

In order to determine whether both cognitive and somatic pre-sleep arousal were associated with reduced sleep quality in the online dataset, we used Spearman’s Rank Correlation Coefficients. In the online dataset, cognitive pre-sleep arousal (r_s_(100) = 0.665, *p* <.001; Fig. 2e) and somatic pre-sleep arousal (r_s_(99) = 0.492, *p* <.001; Fig. 2f) were significantly positively correlated with ISI scores (again, the strength of the associations did not significantly differ from one another; *p* =.102). For the replication dataset, we also found that cognitive pre-sleep arousal (r_s_(95) = 0.423, *p* <.001; Fig. 2e) and somatic pre-sleep arousal (r_s_(95) = 0.290, *p* =.004; Fig. 2f) were significantly positively correlated with ISI scores (the strength of the associations did not significantly differ; *p* =.298).

In the online dataset, both cognitive pre-sleep arousal (r_s_(82) = 0.410, *p* <.001; Fig. 2g) and somatic pre-sleep arousal (r_s_(82) = 0.399, *p* <.001; Fig. 2h) were significantly positively correlated with PSQI scores. Here too we assessed the difference in strength of these correlations using Fisher’s r to z transformation but found no significant difference (*p* =.942). However, these results did not replicate in the second dataset, where neither cognitive pre-sleep arousal (r_s_(92) = 0.087, *p* =.411; Fig. 2f) nor somatic pre-sleep arousal (r_s_(92) = 0.108, *p* =.307; Fig. 2h) were significantly positively correlated with PSQI scores. When controlling for the effect of sex, no difference was found in the outcomes of correlation tests (see supplementary Tables 1 and 2).

### Pre-sleep arousal mediates the relationship between ASRS and ISI

**Fig. 3:**
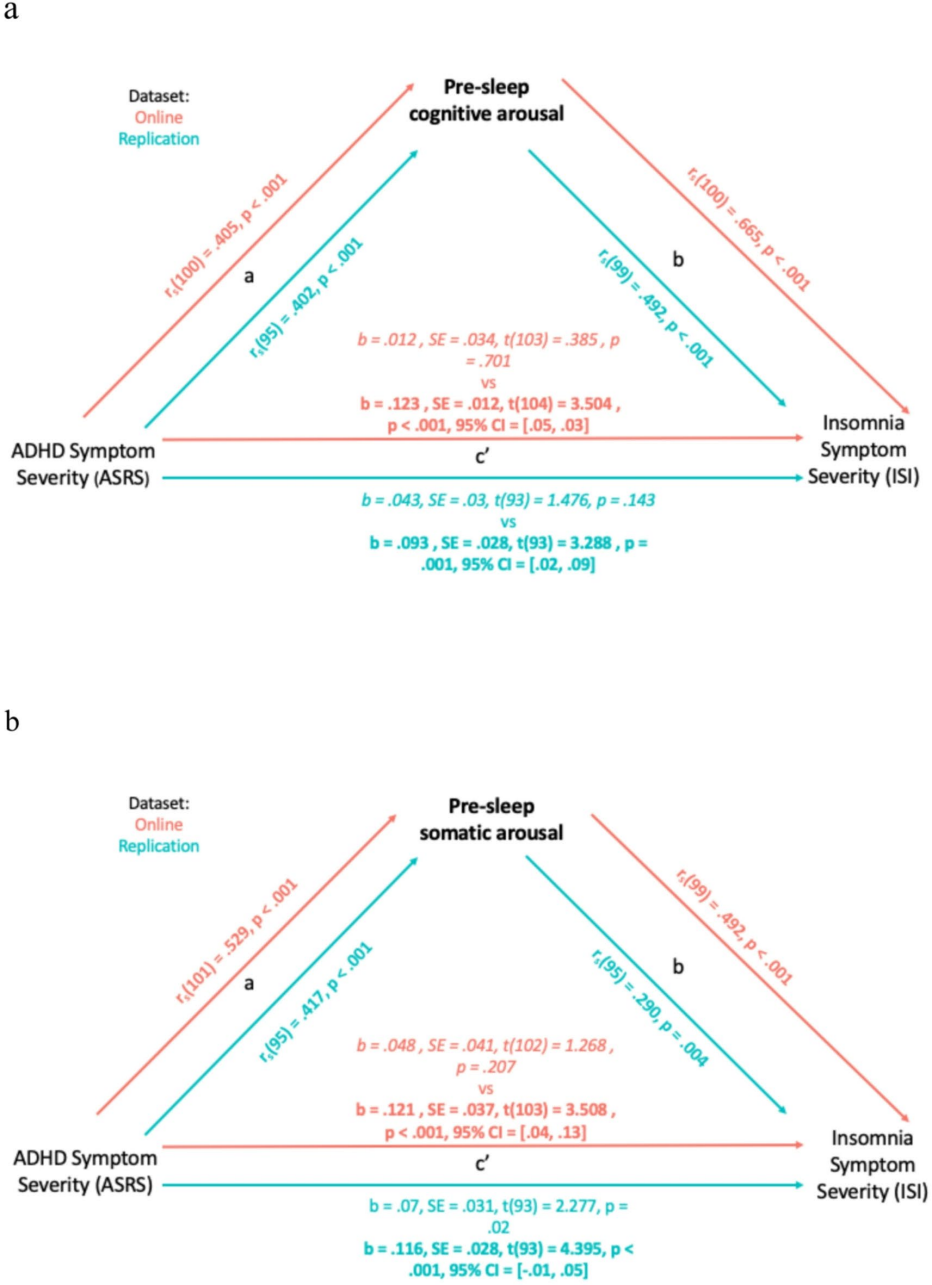
Figures depicting the mediation effect of (**a**) cognitive pre-sleep arousal and (**b**) somatic pre-sleep arousal on the relationship between ADHD symptom severity and Insomnia symptoms severity for the online (red) and replication (blue) datasets.

After verifying the relationship between ADHD symptoms severity and insomnia symptoms severity, and finding a relationship between both of these and pre-sleep arousal, we turned to investigate the possible role of pre-sleep arousal as a mediator, including the measures that showed correlations with each other: ASRS for ADHD symptom severity, ISI for sleep quality, and PSAS for pre-sleep arousal. Specifically, we investigated whether pre-sleep arousal mediates the relationship between ASRS and ISI scores according to the models (illustrated in Fig. 3), where c’ denotes the direct effect of ASRS on ISI whilst the a and b paths elucidate how the relationship between ASRS and ISI is mediated by pre-sleep arousal. These analyses were performed twice, once using pre-sleep cognitive arousal as a mediator and once using pre-sleep somatic arousal.

#### Analysis 1: Pre-sleep cognitive arousal as a mediator

For the online and replication datasets, the mediation analysis revealed a significant indirect effect of ADHD symptom severity on insomnia symptom severity through cognitive pre-sleep arousal. For both datasets, the total effect of ADHD symptom severity and insomnia symptom severity was significant (online dataset: b = 0.123, SE = 0.012, t(104) = 3.504, *p* <.001; replication dataset: b = 0.093, SE = 0.028, t(93) = 3.288, *p* =.001). Again, for both datasets, when controlling for cognitive pre-sleep arousal, the direct effect of ADHD symptom severity on insomnia symptom severity was no longer significant (online dataset: b = 0.012, SE = 0.034, t(103) = 0.385, *p* =.701; replication dataset: b = 0.043, SE = 0.03, t(93) = 1.476, *p* =.143). For both datasets the indirect effect was significant (online dataset: b = 0.099, SE = 0.028, *p* <.001, 95% CI [0.03, 0.05]; replication dataset: b = 0.05, SE = 0.029, *p* <.001, 95% CI [0.02, 0.09]). Since the confidence intervals do not include zero, we conclude that the indirect effects are statistically significant. This indicates that cognitive pre-sleep arousal mediates the relationship between ADHD symptom severity and insomnia symptom severity for both datasets. For the online dataset, 80% of the total effect was mediated (RIT = 0.80), with the indirect effect being over 8 times larger than the direct effect (RID = 8.25). In the replication dataset, 54% of the total effect was mediated (RIT = 0.54), and the indirect effect was slightly larger than the direct effect (RID = 1.16).

#### Analysis 2: Pre-sleep somatic arousal as a mediator

For the online dataset the mediation analysis revealed a significant indirect effect of ADHD symptom severity on insomnia symptom severity through somatic pre-sleep arousal. However, for the replication dataset, it did not. For both datasets the total effect of ADHD symptom severity on insomnia symptom severity was significant (online dataset: b = 0.121, SE = 0.037, t(103) = 3.508, *p* <.001; replication dataset: b = 0.116, SE = 0.028, t(93) = 4.395, *p* <.001). For the online dataset, when controlling for somatic pre-sleep arousal, the direct effect of ADHD symptom severity on insomnia symptom severity was no longer significant (b = 0.048, SE = 0.041, t(102) = 1.268, *p* =.207). However, for the replication dataset, when controlling for the effect of somatic pre-sleep arousal, ADHD symptom severity still significantly predicted insomnia symptom severity (b = 0.07, SE = 0.031, t(93) = 2.277, *p* =.02). For the online dataset, the indirect effect was significant (b = 0.078, SE = 0.023, *p* <.001), with 95% bootstrap confidence interval of [0.04, 0.13]. Since the confidence interval does not include zero, we conclude that the indirect effect is statistically significant. This indicates that somatic pre-sleep arousal mediates the relationship between ADHD symptom severity and insomnia symptom severity. For the online dataset, 64% of the total effect was mediated (RIT = 0.64), with the indirect effect being approximately 1.63 times larger than the direct effect (RID = 1.63). However, for the replication dataset, the indirect effect was not significant (b = 0.023, SE = 0.014, *p* <.001), with 95% bootstrap confidence interval of [−0.01, 0.05], crossing 0. Thus, while somatic pre-sleep arousal mediated the relationship between ADHD symptom severity and insomnia symptom in the online dataset, this finding did not replicate in the in-person dataset.

### The link between pre-sleep arousal and sleep onset latency

In order to determine which specific pre-sleep arousal content is most associated with poorer sleep initiation, we ran a stepwise regression analysis in the online dataset, to model which cognitive and somatic pre-sleep arousal components best predicted subjective sleep onset-latency (PSQI component 2). Two pre-sleep arousal questionnaires (PSAS and GCTI) were included to ensure the most comprehensive analysis of pre-sleep arousal components possible. The final model included GCTI question 21 (thoughts about “your personal life”: *r* =.290, *p* =.002) and PSAS question 13 from the cognitive subscale (“being mentally alert, active”: *r* =.316, *p* <.001) as significant predictors of sleep onset latency (F(2, 109) = 7.547, *p* <.001, R^2^ = 0.106). As GCTI data was not collected for the replication dataset this analysis was not repeated.

### Greater ADHD symptom severity is associated with the pre-sleep predictors of sleep onset latency

Finally, we assessed whether the two specific pre-sleep arousal components implicated in increased sleep-onset latency were also more commonly expressed in those with greater ADHD symptom severity, and whether they could explain increased sleep-onset latency in the condition. Thus, we assessed the relationships between ADHD symptom severity and the two pre-sleep arousal components using Spearman’s Rank Correlational Coefficients. We found that ADHD symptom severity was significantly positively associated with pre-sleep thoughts about “your personal life” (r_s_(104) = 0.355, *p* <.001) and “being mentally alert, active” (r_s_(104) = 0.341, *p* <.001).

## Discussion

In the current study we focused on a possible underlying mechanism – pre-sleep arousal – that may contribute to the high prevalence of sleep initiation problems in individuals experiencing high ADHD traits. As predicted, we found that higher ADHD traits were associated with greater insomnia symptom severity (although not with overall sleep quality as measured by PSQI). This association was robust and replicated across both the online and the replication datasets. These findings align with previous investigations demonstrating a relationship between ADHD symptom severity and sleep quality^47,48^. Furthermore, we found that pre-sleep arousal mediated the association between ADHD symptoms and insomnia symptoms. While this was evident for both somatic and cognitive pre-sleep arousal in the online dataset, in the replication dataset only cognitive pre-sleep arousal mediated the link between ADHD and insomnia symptomatology. Our inclusion of pre-sleep arousal metrics and the mediation analysis point to the critical role pre-sleep arousal is likely playing in driving sleep quality problems (and symptoms of insomnia more specifically) in ADHD.

Our results also showed that greater pre-sleep arousal (both somatic and cognitive) was associated with greater ADHD symptom severity as well as increased insomnia symptom severity. Unlike what has been found in insomnia patients^19^ the differences in associative strength between somatic and cognitive arousal with ADHD symptom severity were not significant. However, regression analyses, implemented as part of the mediation analyses, suggest that the effect of cognitive pre-sleep arousal is more robust than its somatic counter-part. For the online dataset both pre-sleep cognitive and somatic arousal were significantly linked with insomnia symptom severity, but only the link involving pre-sleep cognitive arousal was replicated in the replication dataset. This would be in line with previous findings in insomnia and other sleep problems, which suggest that cognitive pre-sleep arousal has a stronger effect on sleep difficulties than somatic pre-sleep arousal^19,49–51^. The reasons behind the disparity in effect on sleep difficulties by cognitive and somatic pre-sleep arousal remain unclear and warrants further investigation in future research, but they may point to the important role cognitive processes could play in ADHD symptomology and sleep problems.

As our results implicate a role for pre-sleep arousal in the relationship between ADHD symptom severity and insomnia symptom severity, we investigated which specific pre-sleep arousal contents best predicted increased sleep-onset latency. We chose sleep-onset latency from the PSQI as the variable to be predicted, as it is likely that this is the component of sleep that would be most impacted by pre-sleep arousal. We found that the combination of pre-sleep thoughts about “your personal life” and “being mentally alert/active” was a significant predictor of sleep onset latency. Interestingly, these specific pre-sleep arousal components were also significantly associated with ADHD symptom severity. This provides a shared theme of pre-sleep arousal between increased ADHD symptomatology and increased sleep-onset latency, perhaps providing a mechanism for the two to relate to one another. Future research should aim to confirm this shared relationship, such as using brain stimulation to target regions involved in pre-sleep arousal to determine its effect on ADHD symptoms and sleep onset latency, as well as assessing sleep onset latency more objectively using methods such as polysomnography.

A main limitation of the study is that because of the data we collected, we were not able to determine the directionality of the associations. One possible explanation for the association between ADHD symptoms and sleep issues is that ADHD symptoms cause sleep problems, but it may also be the case that sleep problems are causing, or more likely exacerbating, ADHD symptoms. There is some evidence which suggests that poor sleep can result in ADHD-like symptoms in non-ADHD individuals, for example impeding both sustained attention and response inhibition^23–27^. Likely the reality is a mixture of both, with ADHD symptoms exacerbating sleep issues and sleep issues exacerbating ADHD symptoms. If sleep problems are exacerbating ADHD symptoms, their treatment may provide a way of reducing ADHD symptomatology. For example, sleep hygiene interventions, which have been shown to be effective in alleviating sleep problems in ADHD^52^ have led to improvements in cognitive processes which are effected by ADHD, such as planning and attention^53^. Similarly, the directionality of pre-sleep arousal is not determined in the current research. Whilst we confirm a mediating role of pre-sleep arousal in the relationship between ADHD symptoms and sleep problems, our research is not able to determine whether pre-sleep arousal is an effect of ADHD symptoms, of impeded sleep or both. Future longitudinal and interventional studies will be needed to resolve this ambiguity.

While the causal relationships between ADHD symptoms, pre-sleep arousal and sleep problems will need to be more fully investigated, the relationships we have identified provide a basis for thinking about future interventions. In the presence of a potentially complex causal web, interventions targeting sleep may provide a non-pharmacological way of perturbing the system. Sleep problems can have both biological and behavioural causes. For instance, an individual’s innate circadian rhythm is largely driven by activity in the suprachiasmatic nucleus of the hypothalamus (see van Esseveldt et al., 2000 for review; ^54^) and is reliant on the light-dark cycle and CLOCK, PER and BMAL genes^55–57^. However, it can be externally altered. For example, bright light exposure at night, such as from a phone or television, can delay circadian phase^58^ whilst bright light exposure in the morning can advance it^59^. Delays to feeding can also alter CLOCK gene expression^60^ and timing of exercise can also impact circadian rhythm^61^. Thus, as both biological and external factors can impact sleep, treatment for sleep issues can also take a more biological or more behavioural approach.

Due to the relationship between ADHD and sleep and the suggestion of a role for pre-sleep arousal in this relationship, we would argue that targeting pre-sleep arousal provides a potential treatment route for ADHD. Behavioural approaches to sleep improvement have been shown to be effective previously. For example, Cognitive Behavioural Training has been shown to reduce pre-sleep cognitive arousal in people with insomnia^62^. Sleep hygiene training, which focuses on teaching healthy bedtime habits but does not specifically target pre-sleep arousal, has also been shown to improve sleep problems in children with ADHD^63^ who have poorer baseline sleep hygiene than their neurotypical peers^5^. A recent meta-analysis reported that, out of 16 studies examined, 15 reported sleep hygiene interventions improving sleep problems in ADHD children, with improvements including sleep-onset latency, daytime sleepiness and total sleep duration^52^. Implicating pre-sleep arousal as a target for intervention may also suggest a focus on meditation and mindfulness, with this approach already showing promise in relieving sleep issues and specifically reducing pre-sleep cognitive arousal in insomniacs^64–67^.

In terms of biological approaches to treatment, the implication of pre-sleep thoughts surrounding “your personal life” and “being mentally alert/active” provide a framework for understanding which neuronal networks and areas may be involved in heightened pre-sleep arousal in ADHD. These might then be potential targets for treatment, via neurostimulation using methods such as transcranial magnetic stimulation (TMS), rather than the currently more common pharmacological methods (e.g., melatonin tablets). A network which may underpin cognitive pre-sleep arousal is the Default-Mode Network (DMN). The DMN is believed to be the major network which underpins internal mental activity^68^. In ADHD, its activity appears dysregulated and its functional connectivity is sometimes disturbed, leading to hyperactivation which has been hypothesised to cause excessive mind-wandering in the disorder^69,70^. Within the DMN, a region which may be related to pre-sleep thoughts is the retrosplenial cortex (RSC). The RSC is a node of the medial temporal subsystem of the DMN – a system which is reliably activated by thoughts about one’s past and future^68,71–74^. The RSC itself has been demonstrated to be activated by spontaneous autobiographical thoughts during resting state scans^75^. Interestingly, this region has also been implicated in the regulation of neurotypical sleep; it has been suggested to play a key role in consciousness and it has been found to decrease its overall contribution to DMN activity during the descent to sleep^76,77^. In addition, the RSC has been implicated in ADHD as it has been found to have a reduced volume in the disorder when compared to neurotypicals^78^. By shedding light on the specific pre-sleep thought contents which most affect ADHD-related sleep problems, our findings enable future research into potential treatments for ADHD-related sleep problems to be more finely targeted.

It is worth noting that the current research investigated ADHD symptoms, but not an ADHD population. Although our approach addresses the issue of continuous rather than categorical psychopathological symptoms^31,32^ and a wide range of our participants’ ASRS scores would be screened positively for ADHD, it remains to be seen whether our findings would hold in a clinical sample. It is also important to investigate the effect that ADHD medications may have on our findings, as these treatments typically aim to reduce arousal in patients. This may also reduce pre-sleep arousal and alter its mediating effect on ADHD symptoms and sleep problems, but can also cause sleep difficulties^79^. This highlights the benefits of identifying the relationships in the absence of medications, in a non-clinical sample, prior to generalizing to diagnosed patients.

Lastly, we acknowledge that the internal consistency of the ASRS in the replication dataset was relatively low. Previous research suggests that this is common, as the ASRS measures ADHD symptomatology across two distinct dimensions: hyperactivity and impulsivity. Since these dimensions capture different behavioural tendencies, the overall internal consistency of the scale may be lower than that of unidimensional measures, where all items assess a single construct. The inclusion of varied symptom profiles within a single measure can reduce inter-item correlations, leading to a lower Cronbach’s alpha^44^.

In conclusion, our results have highlighted a significant relationship between both cognitive and somatic pre-sleep arousal with ADHD symptom severity and insomnia symptom severity. We argue for a mediating role for these two forms of pre-sleep arousal in the relationship between ADHD and insomnia symptom severity and suggest that the effect of cognitive pre-sleep arousal is more robust than its somatic counterpart. Our findings also highlight a specific role for increased pre-sleep thoughts about “your personal life” and “being mentally alert/active” in sleep-onset latency. Our main finding is the mediating role that pre-sleep arousal plays in the relationship between ADHD traits and sleep quality. To the best of our knowledge, this is a novel contribution that provides a more detailed and nuanced understanding of how and why sleep is disrupted in relation to ADHD. While our study was not based on clinical samples, ADHD is increasingly viewed as existing on a continuum. This suggests that the relationships observed in our study likely extend to those with a clinical diagnosis, with no fundamental differences in underlying mechanisms. Identifying pre-sleep arousal as the specific aspect relating ADHD traits to sleep difficulties highlights a potential target for future interventions aimed at mitigating sleep disturbances in this population. Additionally, it raises important questions about the neurobiological processes occurring during sleep onset that may be specifically impaired in individuals with high ADHD traits. As such, our study not only provides new insights into the link between ADHD and sleep problems but also opens avenues for both basic and interventional research. This work could help direct future treatments of sleep issues by focusing on the role of pre-sleep arousal in ADHD-related sleep problems.

## Electronic supplementary material

Below is the link to the electronic supplementary material.

Supplementary Material 1

## Data Availability

The data that support the findings of this study are available from the corresponding author, [DS], upon reasonable request.

## Declarations

### Permission to reproduce material from other sources statement

Permission to reproduce material from other sources was obtained where applicable.

## References

[1] Faraone, S. V., Biederman, J. & Mick, E. The age-dependent decline of attention deficit hyperactivity disorder: a meta-analysis of follow-up studies. *Psychol. Med.* **36** (2), 159–165. 10.1017/S003329170500471X (2006). 16420712 10.1017/S003329170500471X

[2] Simon, V., Czobor, P., Bálint, S., Mészáros, Á. & Bitter, I. Prevalence and correlates of adult attention-deficit hyperactivity disorder: meta-analysis. *Br. J. Psychiatry*. **194** (3), 204–211. 10.1192/bjp.bp.107.048827 (2009). 19252145 10.1192/bjp.bp.107.048827

[3] Kooij, J. J., Aeckerlin, L. P. & Buitelaar, J. K. [Functioning, comorbidity and treatment of 141 adults with attention deficit hyperactivity disorder (ADHD) at a psychiatric outpatient department]. *Ned Tijdschr Geneeskd*. **145** (31), 1498–1501 (2001). 11512422

[4] Mindell, J. A., Meltzer, L. J., Carskadon, M. A. & Chervin, R. D. Developmental aspects of sleep hygiene: findings from the 2004 National sleep foundation sleep in America poll. *Sleep. Med.* **10** (7), 771–779. 10.1016/j.sleep.2008.07.016 (2009). 19285450 10.1016/j.sleep.2008.07.016

[5] van der Heijden, K. B., Stoffelsen, R. J., Popma, A. & Swaab, H. Sleep, chronotype, and sleep hygiene in children with attention-deficit/hyperactivity disorder, autism spectrum disorder, and controls. *Eur. Child. Adolesc. Psychiatry*. **27** (1), 99–111. 10.1007/s00787-017-1025-8 (2018). 28689312 10.1007/s00787-017-1025-8 PMC5799342

[6] De Crescenzo, F. et al. The use of actigraphy in the monitoring of sleep and activity in ADHD: A meta-analysis. *Sleep. Med. Rev.* **26**, 9–20. 10.1016/j.smrv.2015.04.002 (2016). 26163053 10.1016/j.smrv.2015.04.002

[7] Díaz-Román, A., Mitchell, R. & Cortese, S. Sleep in adults with ADHD: systematic review and meta-analysis of subjective and objective studies. *Neurosci. Biobehav Rev.* **89**, 61–71. 10.1016/j.neubiorev.2018.02.014 (2018). 29477617 10.1016/j.neubiorev.2018.02.014

[8] Gruber, R., Sadeh, A. & Raviv, A. Instability of sleep patterns in children with Attention-Deficit/Hyperactivity disorder. *J. Am. Acad. Child. Adolesc. Psychiatry*. **39** (4), 495–501. 10.1097/00004583-200004000-00019 (2000). 10761352 10.1097/00004583-200004000-00019

[9] Owens, J. et al. Subjective and objective measures of sleep in children with attention-deficit/hyperactivity disorder. *Sleep. Med.* **10** (4), 446–456. 10.1016/j.sleep.2008.03.013 (2009). 18693137 10.1016/j.sleep.2008.03.013

[10] Van Der Heijden, K. B., Smits, M. G. & Gunning, W. B. Sleep hygiene and actigraphically evaluated sleep characteristics in children with ADHD and chronic sleep onset insomnia. *J. Sleep. Res.* **15** (1), 55–62. 10.1111/j.1365-2869.2006.00491.x (2006). 16490003 10.1111/j.1365-2869.2006.00491.x

[11] Bae, S. M. et al. Gender difference in the association between adult attention deficit hyperactivity disorder symptoms and morningness–eveningness. *Psychiatry Clin. Neurosci.* **64** (6), 649–651. 10.1111/j.1440-1819.2010.02140.x (2010). 21155167 10.1111/j.1440-1819.2010.02140.x

[12] Baird, A. L., Coogan, A. N., Siddiqui, A., Donev, R. M. & Thome, J. Adult attention-deficit hyperactivity disorder is associated with alterations in circadian rhythms at the behavioural, endocrine and molecular levels. *Mol. Psychiatry*. **17** (10), 988–995. 10.1038/mp.2011.149 (2012). 22105622 10.1038/mp.2011.149

[13] Bijlenga, D. et al. Associations between sleep characteristics, seasonal depressive symptoms, lifestyle, and ADHD symptoms in adults. *J. Atten. Disord*. **17** (3), 261–275. 10.1177/1087054711428965 (2013). 22210799 10.1177/1087054711428965

[14] Caci, H., Bouchez, J. & Baylé, F. J. Inattentive symptoms of ADHD are related to evening orientation. *J. Atten. Disord*. **13** (1), 36–41. 10.1177/1087054708320439 (2009). 19387003 10.1177/1087054708320439

[15] Gruber, R. et al. Contributions of circadian tendencies and behavioral problems to sleep onset problems of children with ADHD. *BMC Psychiatry*. **12** (1), 212. 10.1186/1471-244X-12-212 (2012). 23186226 10.1186/1471-244X-12-212 PMC3534002

[16] Alvaro, P. K., Roberts, R. M. & Harris, J. K. The independent relationships between insomnia, depression, subtypes of anxiety, and chronotype during adolescence. *Sleep. Med.* **15** (8), 934–941. 10.1016/j.sleep.2014.03.019 (2014). 24958244 10.1016/j.sleep.2014.03.019

[17] Owens, J. A., Dearth-Wesley, T., Lewin, D., Gioia, G. & Whitaker, R. C. Self-Regulation and sleep duration, sleepiness, and chronotype in adolescents. *Pediatrics* **138** (6), e20161406. 10.1542/peds.2016-1406 (2016). 27940688 10.1542/peds.2016-1406

[18] Park, S. et al. Prevalence, correlates, and comorbidities of adult ADHD symptoms in korea: results of the Korean epidemiologic catchment area study. *Psychiatry Res.* **186** (2), 378–383. 10.1016/j.psychres.2010.07.047 (2011). 20724004 10.1016/j.psychres.2010.07.047

[19] Lichstein, K. L. & Rosenthal, T. L. Insomniacs’ perceptions of cognitive versus somatic determinants of sleep disturbance. *J. Abnorm. Psychol.* **89**, 105–107. 10.1037/0021-843X.89.1.105 (1980). 7365114 10.1037//0021-843x.89.1.105

[20] Wicklow, A. & Espie, C. A. Intrusive thoughts and their relationship to actigraphic measurement of sleep: towards a cognitive model of insomnia. *Behav. Res. Ther.* **38** (7), 679–693. 10.1016/S0005-7967(99)00136-9 (2000). 10875190 10.1016/s0005-7967(99)00136-9

[21] Gross, R. T. & Borkovec, T. D. Effects of a cognitive intrusion manipulation on the sleep-onset latency of good sleepers. *Behav. Ther.* **13** (1), 112–116. 10.1016/S0005-7894(82)80054-3 (1982).

[22] Wuyts, J. et al. The influence of pre-sleep cognitive arousal on sleep onset processes. *Int. J. Psychophysiol.* **83** (1), 8–15. 10.1016/j.ijpsycho.2011.09.016 (2012). 21963535 10.1016/j.ijpsycho.2011.09.016

[23] Chuah, Y. M. L., Venkatraman, V., Dinges, D. F. & Chee, M. W. L. The neural basis of interindividual variability in inhibitory efficiency after sleep deprivation. *J. Neurosci.* **26** (27), 7156–7162. 10.1523/JNEUROSCI.0906-06.2006 (2006). 16822972 10.1523/JNEUROSCI.0906-06.2006 PMC6673955

[24] Drummond, S. P. A., Paulus, M. P. & Tapert, S. F. Effects of two nights sleep deprivation and two nights recovery sleep on response Inhibition. *J. Sleep. Res.* **15** (3), 261–265. 10.1111/j.1365-2869.2006.00535.x (2006). 16911028 10.1111/j.1365-2869.2006.00535.x

[25] Goel, N., Rao, H., Durmer, J. S. & Dinges, D. F. Neurocognitive consequences of sleep deprivation. *Semin Neurol.* **29** (4), 320–339. 10.1055/s-0029-1237117 (2009). 19742409 10.1055/s-0029-1237117 PMC3564638

[26] Imeraj, L. et al. Altered circadian profiles in attention-deficit/hyperactivity disorder: an integrative review and theoretical framework for future studies. *Neurosci. Biobehav Rev.* **36** (8), 1897–1919. 10.1016/j.neubiorev.2012.04.007 (2012). 22575380 10.1016/j.neubiorev.2012.04.007

[27] Lowe, C. J., Safati, A. & Hall, P. A. The neurocognitive consequences of sleep restriction: A meta-analytic review. *Neurosci. Biobehav Rev.* **80**, 586–604. 10.1016/j.neubiorev.2017.07.010 (2017). 28757454 10.1016/j.neubiorev.2017.07.010

[28] Diamond, A. Executive functions. *Annu. Rev. Psychol.* **64**, 135–168. 10.1146/annurev-psych-113011-143750 (2013). 23020641 10.1146/annurev-psych-113011-143750 PMC4084861

[29] Willcutt, E. G., Doyle, A. E., Nigg, J. T., Faraone, S. V. & Pennington, B. F. Validity of the executive function theory of Attention-Deficit/Hyperactivity disorder: A Meta-Analytic review. *Biol. Psychiatry 1969*. **57** (11), 1336–1346. 10.1016/j.biopsych.2005.02.006 (2005). 10.1016/j.biopsych.2005.02.006 15950006

[30] Wright, L., Lipszyc, J., Dupuis, A., Thayapararajah, S. W. & Schachar, R. Response Inhibition and psychopathology: A Meta-Analysis of Go/No-Go task performance. *J. Abnorm. Psychol. 1965*. **123** (2), 429–439. 10.1037/a0036295 (2014). 10.1037/a0036295 24731074

[31] Caspi, A. & Moffitt, T. E. All for one and one for all: mental disorders in one dimension. *Am. J. Psychiatry*. **175** (9), 831–844. 10.1176/appi.ajp.2018.17121383 (2018). 29621902 10.1176/appi.ajp.2018.17121383 PMC6120790

[32] Kotov, R. et al. The hierarchical taxonomy of psychopathology (HiTOP): A dimensional alternative to traditional nosologies. *J. Abnorm. Psychol.* **126** (4), 454–477. 10.1037/abn0000258 (2017). 28333488 10.1037/abn0000258

[33] Kavakci, O. et al. Prevalence of attention-deficit/hyperactivity disorder and co-morbid disorders among students of Cumhuriyet university. *Eur. J. Psychiatry*. **26** (2), 107–117. 10.4321/S0213-61632012000200004 (2012).

[34] Dougherty, D. M., Marsh, D. M., Moeller, F. G., Chokshi, R. V. & Rosen, V. C. Effects of moderate and high doses of alcohol on attention, impulsivity, discriminability, and response Bias in immediate and delayed memory task performance. *Alcohol Clin. Exp. Res.* **24** (11), 1702–1711. 10.1111/j.1530-0277.2000.tb01972.x (2000). 11104118

[35] Fernández-Serrano, M. J., Pérez-García, M., Schmidt Río-Valle, J. & Verdejo-García, A. Neuropsychological consequences of alcohol and drug abuse on different components of executive functions. *J. Psychopharmacol. (Oxf)*. **24** (9), 1317–1332. 10.1177/0269881109349841 (2010). 10.1177/0269881109349841 20007413

[36] Buysse, D. J., Reynolds, C. F., Monk, T. H., Berman, S. R. & Kupfer, D. J. The Pittsburgh sleep quality index: A new instrument for psychiatric practice and research. *Psychiatry Res.* **28** (2), 193–213. 10.1016/0165-1781(89)90047-4 (1989). 2748771 10.1016/0165-1781(89)90047-4

[37] Mollayeva, T. et al. The Pittsburgh sleep quality index as a screening tool for sleep dysfunction in clinical and non-clinical samples: A systematic review and meta-analysis. *Sleep. Med. Rev.* **25**, 52–73. 10.1016/j.smrv.2015.01.009 (2016). 26163057 10.1016/j.smrv.2015.01.009

[38] Bastien, C. H., Vallières, A. & Morin, C. M. Validation of the insomnia severity index as an outcome measure for insomnia research. *Sleep. Med.* **2** (4), 297–307. 10.1016/S1389-9457(00)00065-4 (2001). 11438246 10.1016/s1389-9457(00)00065-4

[39] Cerri, L. Q. et al. Insomnia severity index: A reliability generalisation meta-analysis. *J. Sleep. Res.* **32** (4), e13835. 10.1111/jsr.13835 (2023). 36737257 10.1111/jsr.13835

[40] Nicassio, P. M., Mendlowitz, D. R., Fussell, J. J. & Petras, L. The phenomenology of the pre-sleep state: the development of the pre-sleep arousal scale. *Behav. Res. Ther.* **23** (3), 263–271. 10.1016/0005-7967(85)90004-X (1985). 4004706 10.1016/0005-7967(85)90004-x

[41] Correia, I. L. et al. A reliability generalization meta-analysis of the internal consistency and test-retest reliability of the pre-sleep arousal scale (PSAS). *Sleep. Med.* **126**, 290–299. 10.1016/j.sleep.2024.12.030 (2025). 39736256 10.1016/j.sleep.2024.12.030

[42] Harvey, K. J. & Espie, C. A. Development and preliminary validation of the Glasgow content of thoughts inventory (GCTI): A new measure for the assessment of pre-sleep cognitive activity. *Br. J. Clin. Psychol.* **43** (4), 409–420. 10.1348/0144665042388900 (2004). 15530211 10.1348/0144665042388900

[43] Kessler, R. C. et al. The world health organization adult ADHD self-report scale (ASRS): a short screening scale for use in the general population. *Psychol. Med.* **35** (2), 245–256. 10.1017/S0033291704002892 (2005). 15841682 10.1017/s0033291704002892

[44] Kessler, R. C. et al. Validity of the world health organization adult ADHD Self-Report scale (ASRS) screener in a representative sample of health plan members. *Int. J. Methods Psychiatr Res.* **16** (2), 52–65. 10.1002/mpr.208 (2007). 17623385 10.1002/mpr.208 PMC2044504

[45] Shapiro, S. S. & Wilk, M. B. An analysis of variance test for normality (Complete Samples). *Biometrika* **52** (3/4), 591–611. 10.2307/2333709 (1965).

[46] Benjamini, Y. & Hochberg, Y. Controlling the false discovery rate: A practical and powerful approach to multiple testing. *J. R Stat. Soc. Ser. B Methodol.* **57** (1), 289–300. 10.1111/j.2517-6161.1995.tb02031.x (1995).

[47] McGowan, N. M., Voinescu, B. I. & Coogan, A. N. Sleep quality, chronotype and social jetlag differentially associate with symptoms of attention deficit hyperactivity disorder in adults. *Chronobiol Int.* **33** (10), 1433–1443. 10.1080/07420528.2016.1208214 (2016). 27668457 10.1080/07420528.2016.1208214

[48] Uygur, Ö. F. & Bahar, A. The relationship between attention deficit hyperactivity disorder symptoms and bedtime procrastination. *J. Contemp. Med.* **13** (2), 241–246. 10.16899/jcm.1242778 (2023).

[49] Alfano, C. A., Pina, A. A., Zerr, A. A. & Villalta, I. K. Pre-Sleep arousal and sleep problems of Anxiety-Disordered youth. *Child. Psychiatry Hum. Dev.* **41** (2), 156–167. 10.1007/s10578-009-0158-5 (2010). 19680805 10.1007/s10578-009-0158-5 PMC2818382

[50] Gregory, A. M., Willis, T. A., Wiggs, L., & Harvey, A. G. STEPS team. Presleep arousal and sleep disturbances in children. *Sleep* **31** (12), 1745–1747. 10.1093/sleep/31.12.1745 (2008). 10.1093/sleep/31.12.1745 PMC2603482 19090331

[51] Puzino, K., Frye, S. S., LaGrotte, C. A., Vgontzas, A. N. & Fernandez-Mendoza, J. Am I (hyper)aroused or anxious? Clinical significance of pre-sleep somatic arousal in young adults. *J. Sleep. Res.* **28** (4), e12829. 10.1111/jsr.12829 (2019). 30714242 10.1111/jsr.12829

[52] Nikles, J. et al. A systematic review of the effectiveness of sleep hygiene in children with ADHD. *Psychol. Health Med.* **25** (4), 497–518. 10.1080/13548506.2020.1732431 (2020). 32204604 10.1080/13548506.2020.1732431

[53] de Almondes, K. M., Leonardo, M. E. M. & Moreira, A. M. S. Effects of a cognitive training program and sleep hygiene for executive functions and sleep quality in healthy elderly. *Dement. Neuropsychol.* **11** (1), 69–78. 10.1590/1980-57642016dn11-010011 (2017). 29213496 10.1590/1980-57642016dn11-010011 PMC5619217

[54] van Esseveldt, L., K., Lehman, E., Boer, M. N. & () The Suprachiasmatic nucleus and the circadian time-keeping system revisited. *Brain Res. Rev.* **33** (1), 34–77. 10.1016/S0165-0173(00)00025-4 (2000). 10967353 10.1016/s0165-0173(00)00025-4

[55] Kim, M., de la Peña, J. B., Cheong, J. H. & Kim, H. J. Neurobiological functions of the period circadian clock 2 gene, Per2. *Biomol. Ther.* **26** (4), 358–367. 10.4062/biomolther.2017.131 (2018). 10.4062/biomolther.2017.131 PMC6029676 29223143

[56] King, D. P. et al. Positional cloning of the mouse circadian clock gene. *Cell* **89** (4), 641–653. 10.1016/S0092-8674(00)80245-7 (1997). 9160755 10.1016/s0092-8674(00)80245-7 PMC3815553

[57] Takahashi, J. S. Transcriptional architecture of the mammalian circadian clock. *Nat. Rev. Genet.* **18** (3), 164–179. 10.1038/nrg.2016.150 (2017). 27990019 10.1038/nrg.2016.150 PMC5501165

[58] Kelly, T. L. et al. Bright light and LEET effects on circadian rhythms, sleep and cognitive performance. *Stress Med.* **13** (4), 251–258. https://doi.org/10.1002/(SICI)1099-1700(199710)13:4-251::AID-SMI750-3.0.CO;2-0 (1997). 11542396 10.1002/(SICI)1099-1700(199710)13:4<251::AID-SMI750>3.0.CO;2-0

[59] Crowley, S. J. & Eastman, C. I. Phase advancing human circadian rhythms with morning bright light, afternoon melatonin, and gradually shifted sleep: can we reduce morning bright-light duration? *Sleep. Med.* **16** (2), 288–297. 10.1016/j.sleep.2014.12.004 (2015). 25620199 10.1016/j.sleep.2014.12.004 PMC4344919

[60] Wehrens, S. M. T. et al. Meal timing regulates the human circadian system. *Curr. Biol.* **27** (12), 1768–1775e3. 10.1016/j.cub.2017.04.059 (2017). 28578930 10.1016/j.cub.2017.04.059 PMC5483233

[61] Youngstedt, S. D., Elliott, J. A. & Kripke, D. F. Human circadian phase–response curves for exercise. *J. Physiol.* **597** (8), 2253–2268. 10.1113/JP276943 (2019). 30784068 10.1113/JP276943 PMC6462487

[62] Espie, C. A. et al. Attribution, cognition and psychopathology in persistent insomnia disorder: outcome and mediation analysis from a randomized placebo-controlled trial of online cognitive behavioural therapy. *Sleep. Med.* **15** (8), 913–917. 10.1016/j.sleep.2014.03.001 (2014). 24791643 10.1016/j.sleep.2014.03.001

[63] Keshavarzi, Z. et al. In a randomized case–control trial with 10-years olds suffering from attention deficit/hyperactivity disorder (ADHD) sleep and psychological functioning improved during a 12-week sleep-training program. *World J. Biol. Psychiatry*. **15** (8), 609–619. 10.3109/15622975.2014.922698 (2014). 24957753 10.3109/15622975.2014.922698

[64] Cincotta, A. L., Gehrman, P., Gooneratne, N. S. & Baime, M. J. The effects of a mindfulness-based stress reduction programme on pre-sleep cognitive arousal and insomnia symptoms: a pilot study. *Stress Health*. **27** (3), e299–e305. 10.1002/smi.1370 (2011).

[65] Huberty, J. et al. A mindfulness meditation mobile app improves depression and anxiety in adults with sleep disturbance: analysis from a randomized controlled trial. *Gen. Hosp. Psychiatry*. **73**, 30–37. 10.1016/j.genhosppsych.2021.09.004 (2021). 34537477 10.1016/j.genhosppsych.2021.09.004

[66] Ong, J. C., Shapiro, S. L. & Manber, R. Combining mindfulness meditation with Cognitive-Behavior therapy for insomnia: A Treatment-Development study. *Behav. Ther.* **39** (2), 171–182. 10.1016/j.beth.2007.07.002 (2008). 18502250 10.1016/j.beth.2007.07.002 PMC3052789

[67] Rusch, H. L. et al. The effect of mindfulness meditation on sleep quality: a systematic review and meta-analysis of randomized controlled trials. *Ann. N Y Acad. Sci.* **1445** (1), 5–16. 10.1111/nyas.13996 (2019). 30575050 10.1111/nyas.13996 PMC6557693

[68] Andrews-Hanna, J. R. The brain’s default network and its adaptive role in internal mentation. *Neurosci. Baltim. Md.* **18** (3), 251–270. 10.1177/1073858411403316 (2012). 10.1177/1073858411403316 PMC3553600 21677128

[69] Bozhilova, N. S., Michelini, G., Kuntsi, J. & Asherson, P. Mind wandering perspective on attention-deficit/hyperactivity disorder. *Neurosci. Biobehav Rev.* **92**, 464–476. 10.1016/j.neubiorev.2018.07.010 (2018). 30036553 10.1016/j.neubiorev.2018.07.010 PMC6525148

[70] Christakou, A. et al. Disorder-specific functional abnormalities during sustained attention in youth with attention deficit hyperactivity disorder (ADHD) and with autism. *Mol. Psychiatry*. **18** (2), 236–244. 10.1038/mp.2011.185 (2013). 22290121 10.1038/mp.2011.185 PMC3554878

[71] Andrews-Hanna, J. R., Smallwood, J. & Spreng, R. N. The default network and self‐generated thought: component processes, dynamic control, and clinical relevance. *Ann. N Y Acad. Sci.* **1316** (1), 29–52. 10.1111/nyas.12360 (2014). 24502540 10.1111/nyas.12360 PMC4039623

[72] Buckner, R. L., Andrews-Hanna, J. R. & Schacter, D. L. The brain’s default network: anatomy, function, and relevance to disease. *Ann. N Y Acad. Sci.* **1124** (1), 1–38. 10.1196/annals.1440.011 (2008). 18400922 10.1196/annals.1440.011

[73] Schacter, D. L., Addis, D. R. & Buckner, R. L. Remembering the past to imagine the future: the prospective brain. *Nat. Rev. Neurosci.* **8** (9), 657–661. 10.1038/nrn2213 (2007). 17700624 10.1038/nrn2213

[74] Yeo, B. T. T. et al. The organization of the human cerebral cortex estimated by intrinsic functional connectivity. *J. Neurophysiol.* **106** (3), 1125–1165. 10.1152/jn.00338.2011 (2011). 21653723 10.1152/jn.00338.2011 PMC3174820

[75] Andrews-Hanna, J. R., Reidler, J. S., Huang, C. & Buckner, R. L. Evidence for the default network’s role in spontaneous cognition. *J. Neurophysiol.* **104** (1), 322–335. 10.1152/jn.00830.2009 (2010). 20463201 10.1152/jn.00830.2009 PMC2904225

[76] Sämann, P. G. et al. Development of the brain’s default mode network from wakefulness to slow wave sleep. *Cereb. Cortex N Y N 1991*. **21** (9), 2082–2093. 10.1093/cercor/bhq295 (2011). 10.1093/cercor/bhq295 21330468

[77] Vogt, B. A. & Laureys, S. Posterior cingulate, precuneal and retrosplenial cortices: cytology and components of the neural network correlates of consciousness. In: (ed Laureys, S.) Progress in Brain Research. Vol 150. The Boundaries of Consciousness: Neurobiology and Neuropathology. Elsevier 205–217. 10.1016/S0079-6123(05)50015-3 (2005). 10.1016/S0079-6123(05)50015-3 PMC2679949 16186025

[78] Overmeyer, S. et al. Distributed grey and white matter deficits in hyperkinetic disorder: MRI evidence for anatomical abnormality in an attentional network. *Psychol. Med.* **31** (8), 1425–1435. 10.1017/s0033291701004706 (2001). 11722157 10.1017/s0033291701004706

[79] Kidwell, K. M., Van Dyk, T. R., Lundahl, A. & Nelson, T. D. Stimulant medications and sleep for youth with ADHD: A Meta-analysis. *Pediatrics* **136** (6), 1144–1153. 10.1542/peds.2015-1708 (2015). 26598454 10.1542/peds.2015-1708

