# Supplementary material for: Pre-sleep arousal as a possible mechanism driving sleep problems in relation to ADHD traits
Source: Sci Rep. 2025 Jul 8;15:24554. doi: 10.1038/s41598-025-09866-3 (PMC12238469; doi:10.1038/s41598-025-09866-3)
Supplement: Supplementary file 1 — Supplementary Material 1 [file 41598_2025_9866_MOESM1_ESM.docx]

| **Table S1.** Partial correlations controlling for sex for the exploratory dataset. | | | | |
| --- | --- | --- | --- | --- |
|  | df | Correlation Coefficient | p |  |
| ASRS Total |  |  |  |  |
| PSQI Total | 78 | .107 | .173 |  |
| ISI Total | 96 | ****.287** | .002 |  |
| PSAS Cognitive | 98 | *****.409** | < .001 |  |
| PSAS Somatic | 97 | *****.497** | < .001 |  |
| PSAS Cognitive |  |  |  |  |
| PSQI Total | 79 | *****.467** | < .001 |  |
| ISI Total | 97 | *****.656** | < .001 |  |
| PSAS Somatic |  |  |  |  |
| PSQI Total | 79 | *****.370** | < .001 |  |
| ISI Total | 96 | *****.463** | < .001 |  |
| Note: * p < .05, ** p < .01, *** p < .001 | | | |  |

| **Table S2.** Partial correlations controlling for sex for the replication dataset. | | | | |
| --- | --- | --- | --- | --- |
|  | df | Correlation Coefficient | p |  |
| ASRS Total |  |  |  |  |
| PSQI Total | 85 | .065 | .274 |  |
| ISI Total | 88 | ****.305** | .002 |  |
| PSAS Cognitive | 87 | *****.440** | < .001 |  |
| PSAS Somatic | 87 | *****.423** | < .001 |  |
| PSAS Cognitive |  |  |  |  |
| PSQI Total | 85 | .043 | .347 |  |
| ISI Total | 87 | *****.462** | < .001 |  |
| PSAS Somatic |  |  |  |  |
| PSQI Total | 85 | .064 | .278 |  |
| ISI Total | 87 | ****.298** | .002 |  |
| Note: * p < .05, ** p < .01, *** p < .001 | | | |  |
